# Breast cancer and associated factors: a review

**Published:** 2015

**Authors:** MR Ataollahi, J Sharifi, MR Paknahad, A Paknahad

**Affiliations:** * Noncommunicable Diseases Research Center, Fasa University of Medical Sciences, Fasa, Iran; ** Aja University of Medical Sciences, Tehran, Iran; *** Student Research Committee, Hormozgan University of Medical Sciences, Bandar Abbas, Iran

**Keywords:** cancer, breast cancer, women, prevalence, gene

## Abstract

This article investigated different dimensions of breast cancer and its associated factors. It revealed that breast cancer was and continues to be among the most prevalent and growing malignant diseases among Iranian women in the past four decades. In this article, required information was collected through literature review and keyword (cancer, breast cancer, cell, gene, life quality, women, prevalence, productivity, age, obesity, alcohol, cigarette, menopause, genetic, Cytokine, and mortality) query in credible scientific websites such as SID, Google Scholar, and comprehensive portal of human sciences.

This disease affects all physical, mental, and social aspects of women life. On the other hand, such factors as social and family supports during the illness can reduce its damages. Although, the [exact] etiology of breast cancer is unknown, its associated risk factors were identified. Such factors as aging, history of breast cancer in the family, specific changes in breast(s), gene changes, history of productivity and menopause, lack of physical activity, alcohol consumption, obesity, nutrition, race, and radiation therapy to chest are risk factors of breast cancer.

## Introduction

Increased incidence of cancer in recent years and its impact on different physical, mental, and social dimensions of human life have turned it to a major problem of the century [**1**]. The incidence of this disease in developed countries varies from 1 to 2 percent, with almost 5% yearly increase in less developed countries [**2**]. According to estimates, more than 7 million people globally die from cancer. It is predicted that the number of new cancerous cases rises from 10 to 15 million by 2020 [**3**,**4**]. Meanwhile, breast cancer is the most prevalent type of malignant neoplasms among women [**5**] with more than one million new cases per year [**6**]. In Iran, breast cancer accounts for the major type of cancer among women with the incidence of 21.4 [**7**], or 32% [**8**]. Breast cancer is the most common type of cancer among women in the US with the incidence rate of 12.5%. The risk of an individual dying from breast cancer is 1-in-35 [**9**]. At present, the chance of developing breast cancer over lifespan is 12% (1-in-8) in the United States [**10**]. Regarding the importance of this issue, this study sought to investigate breast cancer and its associated factors.

**Cancer and quality of life**

The World Health Organization (WHO) defines the quality of life as an individual's perception of his/her position in life in the context of the culture and value systems in which he/she lives and in relation to his/her goals, expectations, standards, and concerns [**11**]. Cancer affects patients’ quality of life to varying degrees. The major problems affecting patients' life quality are the mental and emotional impacts of illness, diagnostic and therapeutic measures, stress, pain, depression, and disease consequences on family, marital, and social relations, as well as the induced economic burdens, nutritional issues, and treatment complications [**12**,**13**]. Determination of the quality of life of cancer patients can provide medical staff with a new solution in helping them become independent in performing life affairs under critical and non-critical situations [**14**]. Improvement of quality of life of cancer patients is the primary objective of medical and therapeutic cares. Maximization of job capabilities and improvement of functional status and quality of life of the patients are of the important tasks of health care team [**15**].

**Breast cancer and its etiology**

Breast cancer is the most common type of cancer and the second leading cause of death. This disease is the primary cause of mortality among women aged 45–55 years [**16**], and is the second leading cause of cancer-induced death. The incidence of breast cancer is almost 1-in-8 women, requiring complete tissue removal, chemotherapy, radiotherapy, and hormone therapy most of the time [**17**]. Breast cancer is a type of tissue cancer that mainly involves inner layer of milk glands or lobules, and ducts (tiny tubes that carry the milk) [**18**]. The primary risk factors of cancer include age [**19**], high hormone level [**20**], race, economic status, and iodine deficiency in diet [**21**-**23**]. Breast cancer is a multi-stage disease, in which viruses play a role in one stage of this pathogenic process [**24**]. In general, viruses are involved with different cancer types [**25**].

**Social support and breast cancer**

The incidence of breast cancer is 1-in-9 women over lifespan. There are no accurate statistics on the incidence of the disease in Iran, but studies show that breast cancer is the second prevalent type cancer [**26**]. Breast cancer is among diseases with severe psychological impact, in which the thoughts of death and mastectomy cause fear and anxiety in the patient. A cancer patient goes through various psychological stages in coping with and diagnosing this disease. The world of a woman with cancer dramatically collapses in the blink of an eye. The patient becomes confused and her small hopes fade away to great disappointments. Nobody can deeply understand her feelings [**27**,**28**]; while, she strongly needs support. Studies show that support is a vital and multi-dimensional need that should frequently be provided to clients. Nurses and physicians usually prioritize physical support; whereas, psychological-mental supports are polled as more important than other things by such patients. Researchers in the field have addressed hidden suffering of the afflicted women and analyzed their description of the disease and suffering. They collected different reports of change in life cycle and style, in which various concepts such as transition, transformation, overcoming and exploration of meaning have been defined. The discovered meanings functioned as ways by which the patient obtained the accuracy, truth, balance, and integrity [**29**]. In a qualitative study, Hamilton et al. used the grounded theory methodology to investigate the attitude of men as husband, life partner, father, and caregivers about the breast cancer and chemotherapy of their partners. They used semi-structured guided interviews, in which two major subjects were identified: Concentration on the partner's illness, caring for her, and paying attention to family to maintain its flow [**30**]. In a qualitative study, Landmark and Wall analyzed experience of 10 women newly diagnosed with breast cancer (aged 39-60 years). They aimed to improve nurses’ perception of mechanism of the patients' experience. Results revealed some aspects of their life. These experiences included emotional reactions, physical changes of body, mental image change, feminine identity, main activities, and social network. Understanding these experiences is very important for nurses as supporters of patients during the treatment and improvement process. Nurses should learn this knowledge and use it as much as possible in helping women with breast cancer and their families in gaining access to adaptive methods [**31**]. Regarding the deep impacts of this phenomenon on the patient and her family, and to provide them with appropriate support, putting effort to understand the experience of the involved people is very important. This is because a successful management of them and their family requires a comprehensive understanding of their experience [**32**]. Although medical team members may have acquired experience from their personal and professional life, functioning based on these experience limits the power of thinking and judgment [**33**]. Along with the concept of therapy, it is believed that therapists are required to understand the suffering of all people and even themselves as individuals to be capable of providing services in an emotional and empathic framework [**34**].

**Family and breast cancer**

Breast cancer is of the most important factors that risk physical, mental, and social health of women. Some therapeutic complications affect the patient's self-awareness, self-confidence, and sense of self-worthlessness and -acceptance. Suffering from disease, concerning about family future, fear of death, therapeutic complications, reduced performance, and mental imagery disorder are among factors that impair the mental health of patients with breast cancer [**35**]. To women, the loss of breast means losing feminine identity. In addition, although chemotherapy is an important cancer treatment method, it dramatically affects the quality of life of patients and impairs their physical, mental, social, and spiritual well-being [**36**].

Cancer is a disease that involves the whole family. Different studies have reported disruption in daily life of family caregivers. In a qualitative study, two main concepts were found from the experience of partners: concentration of the partner's illness and caring for her, and concentration on family to maintain it. Some marginal concepts in this study included presence, reliance on medical team, decision-making, and handling financial affairs [**30**]. Chronic disease of a family member dramatically affects the whole family. In such circumstances, several factors including role change, doubt, losing the sense of control, stepping into an unfamiliar environment, economic issues, etc. lead to family crisis [**37**]. According to Landmark and Wall, many women wish that their life patterns become normal, the same as before. This is also true to the whole family and can be seen as an adaptive approach. Women consciously choose activities that bring meaningful experience to their body and spirit. These activities vary from fantasizing to engaging in routine housekeeping duties. In their study, the role of supportive systems has been emphasized. They classified such systems into different categories namely family, other women, as well as institutions and organizations including department of surgery and insurance. The majority of patients find support from relatives. It is a valuable support that encourages women to take up against their illness more seriously [**31**].

Results from another study have shown the salient role of family and doctors for several patients. Many participants have mentioned the valuable role of receiving information and support from specialists. Several patients have reported that they have received romantic care and huge support from their families, helping them in adapting to new situation and returning to life. Partners and children, especially daughters, have been an enormous help. Yet, all patients have not had supportive family and have been even left alone [**38**].

**Religion and breast cancer**

Religion is a positive framework for grasping hidden meaning in disease. In the mentioned study, faith was considered as a powerful resource that alleviates concern and stress, and brings real comfort, which can be effective in adaptation with and return to the life [**39**].

**Cigarette smoking and breast cancer**

Identification of Breast cancer, as the most important cancer in women, and exploring its risk factors have interested researchers for many years [**40**]; however, the role of cigarette has not been considered as a cause until recently [**41**]. Increased incidence of breast cancer parallel with lung cancer in women in recent decades have attracted researchers towards increased rate of female smokers, aiming at finding a similar cause for this ascending trend. It is almost for two decades that researchers have addressed the relationship between breast cancer and cigarette smoking, leading to at least 22 published articles only by the late 80s. Different studies have suggested a weak relationship, lack of relationship, or supportive effect. The emphasis of these articles has been on active cigarette smoking and breast cancer. Investigation into indirect correlation of cigarette smoking with breast cancer has been less undertaken, but has delivered fixed results. Women exposed to cigarette smoke during childhood or married to a cigarette smoker are more prone to breast cancer [**42**,**43**]. In a meta-analysis by Kuder et al. into indirect exposure to cigarette-smoke and the risk of breast cancer, a weak relationship was found; therefore, further studies are required to prove this causal relationship [**44**]. Results of Reynold et al.’s study on 116,544 women showed increased chance of developing breast cancer in cigarette-smokers, corroborating the role of cigarette in breast cancer etiology [**45**]. Rousseau et al. determined the susceptibility of breast tissue through growing and differentiating it [**46**]. Breast cells differentiated from the parts 1 and 2 are susceptible to chemical mutagens that occur before menopause; whereas, those differentiated from the part 3 are mutagen-immune. According to this study, it is supposed that exposure period to breast carcinogens determines susceptibility to carcinogenesis. For example, an early exposure, especially before the first pregnancy, may end in breast cancer, due to genotoxic mechanisms; whereas, the subsequent exposures have protective effects because of anti-estrogenic characteristic of cigarette. However, it should be considered that the duration of cigarette-smoking may neutralizes this effect. As a result, it is very important to determine whether the exposure to cigarette smoke was direct or indirect. The discovered relationship of cigarette-smoking with breast cancer (1984) confirmed this protective effect [**47**]. A study (1990) showed that the relative chance of developing breast cancer in cigarette-smokers versus non-smokers was 1-in-12 in case studies and 1-in-14 in cohort studies [**48**]. The onset of smoking in younger age increases the risk of cancer breast. Women who started smoking at 10-14 years were more prone to breast cancer [**49**]. The risk of breast cancer is higher in women with family history of breast cancer, ovarian cancer, or both [**50**].

**Cancer and genetic factors**

Breast cancer is a highly heterogeneous disease that is developed by mutual impact of genetic risk factors and environmental factors. It leads to progressive aggregation of genetic and epigenetic changes in breast cancer cells. Although epidemiological evidence highlight the presence of risk factors (such as age, obesity, alcohol use, and exposure to estrogen in lifetime), family history of breast cancer is the strongest one. Almost 20% of all breast cancers have family origin, and etiologically are dependent to a specific predisposing gene of that disease [**51**].

**Nutritional factors and breast cancer**

Among the nutritional factors, weight gain and high calorie intake are two causes of breast cancer development. Kopans and Greenwald put that obesity and high BMI in post-menopause increases the risk of breast cancer; whereas, there is not such relationship in pre-menopause women [**52**]. For the first time in 1940, research findings showed that increased use of fat leads to breast tumor in animals [**53**]. Howe and Goodwin reported a positive correlation between high fat intake and the risk of breast cancer [**54**]. Another study reported a positive significant relationship between animal protein intake and the risk of breast cancer [**55**]. In general, the relationship with the risk of breast cancer development is uncertain [**56**]. On the one hand, calorie intake leads to weight gain and obesity; on the other hand, it results in increased height in childhood and preterm menopause. Both factors can establish the context for cancer development in future [**57**].

**BRCA1 and breast cancer**

The main risk factors of non-genetic breast cancer have hormonal origin. For example, gender, the age at menarche and menopause, reproductive history, breast-feeding, and the use of exogenous estrogen (with external origin) can be mentioned. In most cases, non-genetic breast cancer occurs among menopausal women who have high expression of estrogen receptor. Estrogen has at least two main roles in breast cancer development: (1) Estrogen metabolites can mutate or generate DNA-damaging free radicals [**58**], and (2) estrogen can proliferate cells in precancerous and cancerous lesions through its hormonal activity. In addition, since an important part of breast carcinoma is estrogen-receptor-negative (or ER-), other mechanisms are also involved in the development of breast cancer [**59**]. Mutation of BRCA1 raises the risk of breast cancer to 51% and 85% by the age 50 and 70 years, respectively; it also raises the risk of ovarian cancer to 23% and 63% by the age 50 and 70 years, respectively [**60**].

**Immune system and breast cancer**

The immune system is totally able to combat tumors and many immunological parameters applying cytokines for example IL-12 & IFN-γ play major roles in this regard. IL-12 is also the major cytokine responsible for the differentiation of TH1 cells, which are potent producers of IFN-γ, IFN-γ in turn has a powerful enhancing effect on the ability of phagocytes to produce IL-12 as well as having an important role in cellular immune response [**61**]. 

**Breast cancer in Iran**

The incidence of this disease is about 20-in-1000 per year in Iran. Therefore, the probability of new cases with breast cancer is 6,000 (almost 1-in-10), out of 30 million women in the country [**62**]. Although Iran has lower incidence of breast cancer, as compared to other countries, recent increase of this problem has turned it into the most common type of lesion among Iranian women. This cancer affects Iranian women at least one decade earlier than their counterparts in developed countries (more than 30% of the patients are younger than 30 years) [**63**,**64**].

**Methodology**

In this article, required information was collected through literature review and keyword (cancer, breast cancer, cell, gene, life quality, women, prevalence, productivity, age, obesity, alcohol, cigarette, menopause, genetic, Cytokine, and mortality) query in credible scientific websites such as SID, Google Scholar, and comprehensive portal of human sciences.

## Conclusion

Breast cancer was and continues to be among the most prevalent and growing malignant diseases among Iranian women in the past four months. Breast cancer is a disease that involves the patient, family, and community, and wastes many financial and spiritual resources. This cancer is developed in breast tissues including ducts (tiny tubes that carry the milk) and lobules (milk-producing glands). Breast cancer is not gender-specific, but rarely develops in men. Although the exact cause of breast cancer is unknown, specific risk-factors have been identified. Different types of cancer have different risk factors. Some of these risk factors such as cigarette-smoking, alcohol use, and diet can be changed and depend on life style. However, other factors like age, race, gender, and family history are fixed and unchangeable. Having one or more of these risk factors does not necessarily mean infliction.

Although many of these risk factors increase the chance of breast cancer development and progress, its exact mechanism is not clear. It seems that hormone plays a very important role in some types of breast cancer; however, its development and progress mechanisms are not very clear. In general, it can be said that such factors as aging, history of breast cancer development in family, certain changes in breasts, genetic changes, history of productivity and menopause, lack of physical activity, alcohol-use, diet and nutrition, race, and radiation therapy to chest are risk factors of breast cancer.

This disease affects different physical, mental, and social aspects of women life. On the other hand, such factors as social and family supports during the illness can reduce its negative impacts.
